# How can latent trajectories of back pain be translated into defined subgroups?

**DOI:** 10.1186/s12891-017-1644-8

**Published:** 2017-07-03

**Authors:** Alice Kongsted, Lise Hestbæk, Peter Kent

**Affiliations:** 1 Nordic Institute of Chiropractic and Clinical Biomechanics, Campusvej 55, DK-5230 Odense M, Denmark; 2 Department of Sports Science and Clinical Biomechanics, University of Southern Denmark, Campusvej 55, DK-5230 Odense M, Denmark; 3 Department of Physiotherapy and Exercise Science, Curtin University, Perth, Australia

**Keywords:** Classification, Low back pain, Subgroups, Trajectory

## Abstract

**Background:**

Similar types of trajectory patterns have been identified by Latent Class Analyses (LCA) across multiple low back pain (LBP) cohorts, but these patterns are impractical to apply to new cohorts or individual patients. It would be useful to be able to identify trajectory subgroups from descriptive definitions, as a way to apply the same definitions of mutually exclusive subgroups across populations. In this study, we investigated if the course trajectories of two LBP cohorts fitted with previously suggested trajectory subgroup definitions, how distinctly different these subgroups were, and if the subgroup definitions matched with LCA-derived patterns.

**Methods:**

Weekly measures of LBP intensity and frequency during 1 year were available from two clinical cohorts. We applied definitions of 16 possible trajectory subgroups to these observations and calculated the prevalence of the subgroups. The probability of belonging to each of eight LCA-derived patterns was determined within each subgroup. LBP intensity and frequency were described within subgroups and the subgroups of ‘fluctuating’ and ‘episodic’ LBP were compared on clinical characteristics.

**Results:**

All of 1077 observed trajectories fitted with the defined subgroups. ‘Severe episodic LBP’ was the most frequent pattern in both cohorts and ‘ongoing LBP’ was almost non-existing. There was a clear relationship between the defined trajectory subgroups and LCA-derived trajectory patterns, as in most subgroups, all patients had high probabilities of belonging to only one or two of the LCA patterns. The characteristics of the six defined subgroups with minor LBP were very similar. ‘Fluctuating LBP’ subgroups were significantly more distressed, had more intense leg pain, higher levels of activity limitation, and more negative expectations about future LBP than ‘episodic LBP’ subgroups.

**Conclusion:**

Previously suggested definitions of LBP trajectory subgroups could be readily applied to patients’ observed data resulting in subgroups that matched well with LCA-derived trajectory patterns. We suggest that the number of trajectory subgroups can be reduced by merging some subgroups with minor LBP. Stable levels of LBP were almost not observed and we suggest that minor fluctuations in pain intensity might be conceptualised as ‘ongoing LBP’. Lastly, we found clear support for distinguishing between fluctuating and episodic LBP.

## Background

The outcome trajectories of individuals with low back pain (LBP) show diverse patterns, and data-driven analyses have demonstrated that distinct trajectory subgroups exist that not only differ in pain severity but also in their course pattern [1–7]. People with different LBP trajectories also differ on a number of other characteristics and so subgrouping LBP by course trajectories may be helpful as a way to define relatively homogenous phenotypes of ‘non-specific’ LBP [8]. Therefore, there is interest in whether these phenotypes might facilitate better prognostic estimates and more targeted treatment [9, 10].

The data-driven subgrouping of LBP course patterns, which has been primarily conducted using Latent Class Analyses (LCA), has identified broadly similar types of trajectory patterns across multiple LBP cohorts [8]. However, different terminology has been used to describe these patterns, and the ‘latent’ patterns identified by LCA are difficult to directly compare for a number of reasons. One reason is that in LCA, people are not grouped into mutually exclusive groups but instead have a certain probability of belonging to each latent class (albeit they often have a relatively high probability of belonging to only one latent class). Furthermore, the specific trajectory patterns identified can depend on the type of data informing the analyses (e.g. categorical versus continuous variables), the frequency of data collection, and the size and composition of the study sample [11, 12]. Also, latent classes are impractical for clinical situations because the statistical parameters from the LCA model would be needed for application of the derived patterns to individual new patients.

Therefore, it would clearly be useful to be able to identify trajectory subgroups from descriptive definitions that could be easily applied to independent datasets or individual new patients as a way to apply the same definitions of mutually exclusive subgroups across populations. For example, we would need standardised definitions if we were to determine if certain trajectory patterns are more frequent in some populations than others or following particular treatments. Also, it would be very useful to operationally define specific and clearly described trajectory subgroups as a means to facilitating investigations into whether trajectory patterns are clinically useful indicators of relatively homogenous LBP phenotypes.

We participated in a collaborative group that suggested standardising definitions for labelling LCA-derived trajectory patterns in order to provide a common terminology and promote consistency within this research field [8]. These were consensus-based suggestions that captured the general features of LBP trajectory patterns that had been identified across different settings and different methods. The suggested labels described LBP trajectories in terms of pain intensity, pain variation over time, and the early change patterns after initiating care (Table 1).

**Table 1:** Descriptions of the defined trajectory subgroups

Principal dimensions of trajectory subgroups	Terminology for labelling	Suggested definition
*INTENSITY*		
		Mean scores 0–10 Numerical Rating Scale
	*Severe pain*	6–10
	*Moderate pain*	4–5
	*Mild pain*	2–3
	*Minor pain/Recovery* ^*a*^	0–1
*VARIATION*		
	*Ongoing pain*	An individual’s pain intensity stays within mean +/− 1-point (0–10 NRS) Pain reported >4 days per week
	*Fluctuating pain*	Variation in pain intensity exceeds 2 points*, without periods of no pain (0) lasting ≥1 month [21]
	*Episodic pain*	Experiencing more than one period of pain over 1 year separated by periods with no pain (0) lasting ≥1 month
	*Single episode*	Experiencing one episode lasting ≤ 2 weeks within 1 year
*Change pattern* (likely to be most relevant for clinical populations)		
	*Rapidly improving pain*	Marked decrease in pain intensity within 1 month
	*Gradually improving pain*	Marked decrease in pain intensity occurring gradually over more than 1 month
	*Progressing pain*	An overall pattern of increasing pain intensity

^a^Our interpretation of the definition of fluctuations was that the difference between minimum and maximum pain intensity exceeded 2 points

From Kongsted A, Kent P, Axen I, Downie AS, Dunn KM. What have we learned from ten years of trajectory research in low back pain? BMC Musculoskelet Disord 2016; 17: 220

Although these definitions were primarily intended to facilitate a common terminology, it would be very useful if they could also be used to descriptively classify people into pre-defined trajectory subgroups in new data without the need to perform LCA. However, two practical requirements of such a process would be that most people’s observed trajectories would fit into the trajectory definitions when those definitions were applied to new LBP data, and that there was a good classification match between the trajectory patterns identified by LCA and the trajectory definitions. Currently, it is unknown to what extent either is true.

This study was an initial step in establishing useful definitions of trajectory subgroups. By applying the suggested trajectory subgroup definitions to two clinical LBP datasets the objectives of this study were to determine: (1) the prevalence of observed LBP trajectories that fitted into these subgroup definitions, (2) how individuals’ classification into defined trajectory subgroups matched their membership of previously identified LCA-derived trajectory patterns, and (3) how distinctly different the defined subgroups were. On the basis of these results, we then make suggestions about how the trajectory subgroup definitions could be refined.

## Methods

This study is a secondary analysis of data from an observational cohort study that consisted of a sample from general practice and a sample from chiropractic practice. Descriptions of the samples and results from other studies of this cohort have been published [4, 13–15]. Patients were included when seeking care for LBP and were followed weekly for 1 year after inclusion.

**Fig. 1:**
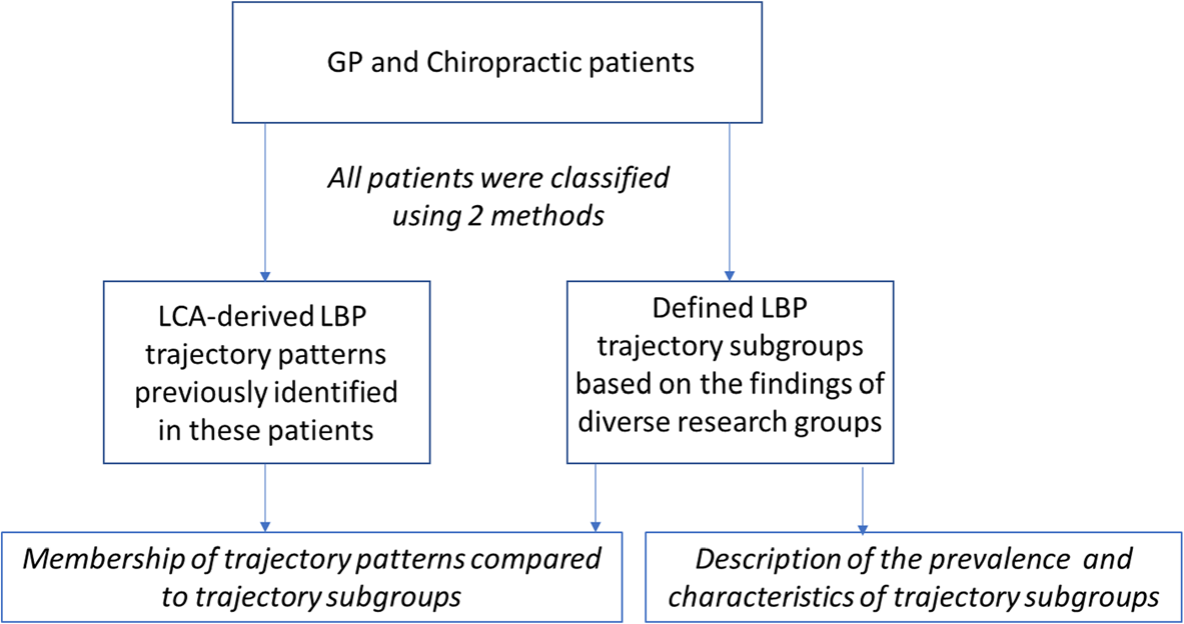
Outline of the study design

In the current study, weekly measures of pain intensity were used to subgroup patients in these samples into trajectory subgroups using previously reported definitions that were based on trajectory patterns observed across a number of studies (hereafter referred to as ‘defined trajectory subgroups’) [8]. This subgrouping was compared to patients’ membership in eight LCA-derived trajectory patterns that had been previously identified within this sample (hereafter called ‘LCA-derived trajectory patterns’) [4] (Fig. 1).

### Participants

Participants were people 18–65 years of age seeking care from general practitioners or chiropractors for non-specific LBP, with or without accompanying leg pain. An exclusion criterion for patients seeking chiropractic care was if they had received treatment for LBP within 3 months prior to the index consultation. The patients received care as usual in these settings and this care was unaffected by inclusion in the study.

A total of 947 chiropractic patients were recruited from 17 chiropractic clinics in the research network of the Nordic Institute for Chiropractic and Clinical Biomechanics between September 2010 and January 2012. From general practice, 251 patients were recruited during a 10-week period during 2011 from 88 general practitioners who were part of a quality development initiative by the Audit Project Odense. During the remainder of 2011 after the audit period, an additional 73 patients were recruited from the 12 general practitioners who had recruited most patients during the audit and 18 other general practitioners known to have an interest in LBP.

### Variables

Descriptive baseline characteristics obtained from patient-completed questionnaires included: number of previous episodes (0, 1–2, 3 or more), duration of current episode (0–2 weeks, 2–4 weeks, 1–3 months, >3 months), typical LBP and leg pain intensity the preceding week on a 0–10 Numeric Rating Scale (0 = no pain; 10 = worst imaginable pain), and distress measured by the Major Depression Inventory (0 = no depressive symptoms; 50 = maximum depressive symptoms) [16].

**Fig. 2:**
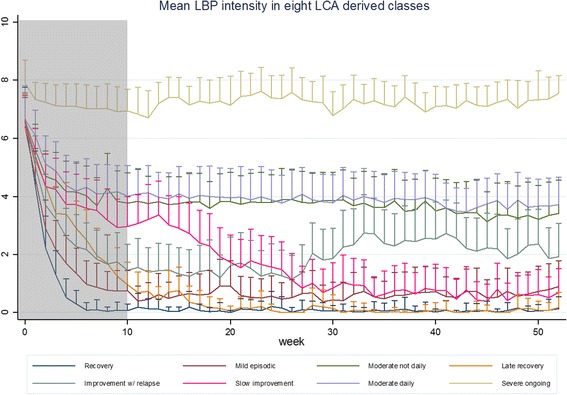
Mean LBP intensity in eight previously identified latent LBP trajectory patterns. In order to initially describe general patterns of LBP in relatively stable trajectory periods, the measures from the first 9 weeks after initiating treatment (grey area) were not included in this study. Bars indicate +½ standard deviation, which were used to enhance the readability by avoiding overlap of graphs

The course of LBP was captured through questions sent weekly by an automated SMS service to participants’ mobile phones over a 12-month period. The questions asked were “How many days did you have low back pain during the last week? (report a number between 0 and 7)” and “How intense was the pain typically on a scale from 0 to 10?” (where 0 = no pain). In this study, we excluded responses from the first 9 weeks of healthcare when pain improved rapidly because we wanted to initially explore the match of the observed data to trajectory definitions of LBP within periods of relatively stable clinical course rather than focusing on the clinical course that closely followed the initiation of treatment (Fig. 2).

Activity limitation at baseline and 3 months later was measured by the Roland Morris Disability Questionnaire (recalculated as proportional scores 0 = no limitation; 100 = maximum disability) [17, 18] .

Patients’ expectations of future LBP were obtained 12 months after baseline using a novel and un-validated question that sought to evaluate patients’ perceptions of the nature of their condition. The participants were asked which of seven LBP scenarios they thought most likely to occur within the next 5 years. The listed scenarios were: *no LBP*, *episodic mild LBP*, *rather constant mild LBP*, *episodic LBP of varying intensity*, *rather constant LBP of varying intensity*, *episodic severe LBP*, or *rather constant severe LBP.*

### Defined LBP trajectory subgroups

The definitions of trajectory subgroups were a descriptive combination of pain intensity (minor/recovery, mild, moderate or severe) and pain variation (ongoing, fluctuating, episodic or single episode) [8]. Episodic LBP was defined as pain reoccurring after a pain-free period of at least 4 weeks based on previously suggested definitions from literature reviews and a modified Delphi process [19, 20]. For clinical populations, the option of describing the initial change pattern (rapidly improving pain, gradually improving pain or progressing pain) was also included in the published subgroup definitions (Table 1). However, in this initial explorative study, we did not include the initial change patterns because we wanted to firstly investigate the potential usefulness of the subgroup definitions in their simplest form.

### LCA-derived LBP trajectory patterns

In a previous study, we performed LCA using weekly measures of LBP intensity and frequency as input to identify characteristic LBP trajectory patterns [4]. We applied 12 different LCA models resulting in slightly different trajectory patterns. For the purpose of the current study, we compared the defined trajectory subgroups to a LCA model that had resulted in eight latent trajectory patterns labelled *recovery*, *late recovery*, *slow improvement*, *mild episodic*, *improvement with relapse*, *moderate ongoing daily*, *moderate ongoing non-daily*, and *severe ongoing* (Fig. 2)*.* Because we did not include data from the first 9 weeks, we considered *recovery* and *late recovery* to be one trajectory pattern. The 8-class model was chosen as a balance between the simplest 5-class model and the most complex 12-class model. The certainty of classifying each individual into these eight trajectory patterns was high (the proportion classified with <0.50 posterior probability of class membership was 0.1%, average posterior probability 0.99).

### Data analyses

Missing values on the weekly pain intensity measures were handled in three steps: (1) missing responses in week 10 (the first week included) were replaced by the equivalent values in week 11 if they were not missing, and similarly, missing responses in week 52 were replaced by the values reported in week 51, (2) one-week and two-week gaps between weeks, where the same pain intensity was reported, were replaced with that same value; (3) participants who after steps 1 and 2 had less than 20 complete responses out of 43 were dropped. Missing values that were still present after these procedures were deemed acceptable.

When classifying participants into defined trajectory subgroups, the definitions were operationalised as described in Table 2. These definitions were used for generating a subgrouping variable that allowed us to automatically allocate people to the defined subgroups based on their weekly NRS scores of pain intensity and weekly measures of number of days with LBP. For example, this subgrouping variable would equal ‘severe ongoing LBP’ if mean LBP intensity across 43 NRS scores was at least 6, the pain intensity did not exceed +/− 1 point from the mean in any week, and for all weeks the frequency was above 4 days per week. Fluctuations were operationalised as pain intensities deviating more than 1 point from either side of the mean, which corresponds to total variations of 2 points. This classification into defined subgroups was performed independently of the previous LCA analysis. The prevalence of people who matched these subgroups within the general practice and the chiropractic cohorts was determined as well as the frequency of non-classification. To describe the extent to which LBP differed across subgroups, the mean LBP intensity and mean number of pain days in weeks when any pain was reported was calculated for each subgroup. This was simply descriptive and no statistical testing of differences was performed.

**Table 2:** Operational definitions used for the previously defined trajectory subgroups

Subgroup label	Intensity	Variation
Severe ongoing pain	Mean intensity > = 6	Intensity stays within +/− 1 of mean value >4 days with LBP per week
Moderate ongoing pain	Mean intensity > = 4 and <6	Intensity stays within +/− 1 of mean value >4 days with LBP per week
Mild ongoing pain	Mean intensity > = 2 and <4	Intensity stays within +/− 1 of mean value >4 days with LBP per week
Minor ongoing pain/ recovery	Mean intensity <2	Stays within +/− 1 of mean value AND - no pain-free 4-weeks periods *or* - always pain = 0
Severe fluctuating pain	Mean intensity > = 6	Difference between mean and minimum or maximum value exceeds 1 No pain-free 4-weeks periods
Moderate fluctuating pain	Mean intensity > = 4 and <6	Difference between mean and minimum or maximum value exceeds 1 No pain-free 4-weeks periods
Mild fluctuating pain	Mean intensity > = 2 and <4	Difference between mean and minimum or maximum value exceeds 1 No pain-free 4-weeks periods
Minor fluctuating pain	Mean intensity <2	Difference between mean and minimum or maximum value exceeds 1 No pain-free 4-weeks periods
Severe episodic pain	Max intensity > = 6	Pain-free periods of min. 4 weeks in a row between weeks with pain. Four weeks or more without pain in the beginning or end of the course does not indicate a new episode.
Moderate episodic pain	Max intensity > = 4 and <6	Pain-free periods of min. 4 weeks in a row between weeks with pain. Four weeks or more without pain in the beginning or end of the course does not indicate a new episode.
Mild episodic pain	Max intensity > = 2 and <4	Pain-free periods of min. 4 weeks in a row between weeks with pain. Four weeks or more without pain in the beginning or end of the course does not indicate a new episode.
Minor episodic pain	Max intensity <2	Pain-free periods of min. 4 weeks in a row, but not always pain = 0. Four weeks or more without pain in the beginning or end of the course does not indicate a new episode.
Severe single episode	Max intensity > = 6	One episode lasting 1–2 weeks (which are not the first or the last week of measurement)
Moderate single episode	Max intensity > = 4 and <6	One episode lasting 1–2 weeks (which are not the first or the last week of measurement)
Mild single episode	Max intensity > = 2 and <4	One episode lasting 1–2 weeks (which are not the first or the last week of measurement)
Minor single episode	Max intensity <2	One episode lasting 1–2 weeks (which are not the first or the last week of measurement)

To compare the defined trajectory subgroups to the LCA-derived trajectory patterns, the average posterior probability of belonging to each of the LCA-derived trajectory patterns was calculated for the people who had been classified within each of the defined trajectory subgroups. The posterior probabilities were obtained from the pre-existing LCA analysis.

To explore the characteristics of observed fluctuations in patients’ pain, we described the proportion of people with any fluctuations (defined as pain intensity deviating more than 1 point from their mean), the number of weeks with a fluctuation, and the size of the observed fluctuations. To describe episodic patterns of pain, we looked at the number and duration of completely pain-free periods. In addition, to explore if there was empirical support in our data for the threshold of a pain-free period of 4 weeks (a recommended threshold defining a break between episodes [19, 20]) being different to pain-free weeks of shorter or longer duration, we described the pain intensity and number of days with LBP that occurred in the first week after pain-free breaks of different durations. We speculated that if there was more intense pain in the week after four or more weeks without pain, as compared to after shorter pain-free periods, this would provide some empirical support that this threshold of pain-free periods could be a particularly useful hallmark in defining a break before a new episode of LBP.

As fluctuating LBP and episodic LBP have been identified as two different trajectory patterns, we were interested in determining if these trajectories actually appeared to be distinctly different subgroups. For that purpose, we compared the clinical characteristics (distress, leg pain, activity limitation and patients’ expectations of future LBP) between people who had been classified in the fluctuating and episodic patterns, stratified by pain intensity levels (severe, moderate, mild, minor). In a sensitivity analysis, we repeated this comparison after changing the definition of ‘episodic’ from requiring 4 weeks or more without pain to 2 weeks or more without pain.

All analyses were performed in STATA 14.2 (StataCorp LP, Texas, USA).

## Results

Baseline data were available from 1240 patients (302 from general practice, 938 from chiropractic practice). From both settings, 13% were excluded due to less than 20 responses out of 43 possible follow-up measurement points, leaving a study cohort of 1077 participants. The baseline characteristics of the study cohort and of those excluded due to missing responses are summarised in Table 3.

**Table 3:** Baseline characteristics of the sample

	General practice sample (*n* = 263)	Chiropractic sample (*n* = 814)	Excluded (*n* = 163)
Female (%)	57%	45%	47%
Age, mean (sd)	45 (11)	43 (11)	44 (13)
Episode duration (%)			
0–2 weeks	39%	63%	55%
2–4 weeks	14%	14%	11%
1–3 months	16%	11%	11%
> 3 months	32%	13%	23%
LBP intensity at baseline (NRS 0–10), median (IQR)	7 (6–8)	7 (5–8)	7 (6–9)
Leg pain intensity at baseline (NRS 0–10), median (IQR)	3 (0–6)	2 (0–4)	2 (0–6)
Previous LBP episodes			
0	15%	16%	18%
1–2	24%	35%	30%
3 or more	61%	49%	52%
RMDQ (0–100), median (IQR)	61 (39–78)	52 (35–70)	61 (39–78)

*IQR*, Interquartile range, *RMDQ* Roland Morris Disability Questionnaire score (0 = no disability; 100 = complete disability). *NRS* Numeric Rating Scale

### Match of data to the defined LBP trajectory subgroups

**Fig. 3:**
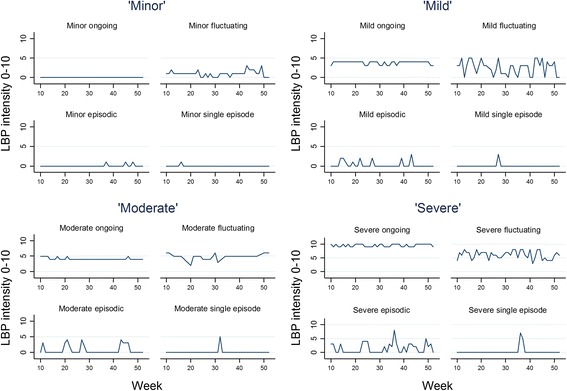
Examples of observed LBP trajectories within each of 16 defined subgroups

All of the cohort could be classified into one of the pre-defined subgroups. An example of an individual trajectory purposefully selected to represent typical trajectories from each subgroup is provided in Fig. 3.

The most common patterns were those described as episodic, with ‘severe episodic LBP’ being the most frequent pattern in both settings (24% and 28%) (Table 4). Within this subgroup, the total number of pain days during 43 weeks ranged from two to 253 days. Following the ‘episodic LBP’ subgroups, the ‘fluctuating LBP’ subgroups were the second most frequent. In general, severe or moderate pain intensities were mostly observed in episodic pain subgroups, mild pain was mostly observed in fluctuating or episodic subgroups, and minor pain tended to present as a stable pattern. Except for the ‘minor ongoing LBP/recovered’ subgroup (16% and 8% in the two settings), the ongoing (stable) subgroups were almost non-existent in this cohort (0.1% to 1% of the samples).

**Table 4:** Distribution of patients in the defined subgroups

Defined Trajectory Subgroup	Prevalence (n) of patients in the chiropractic sample (*n* = 814)	Prevalence (n) of patients in the general practice sample (*n* = 263)	Posterior probability of belonging to each of eight LCA derived patterns^a^	Number of days with pain per week, in weeks with any LBP, mean (SD)	Pain intensity in weeks with any LBP, mean (SD)	Total number of days with LBP during 43 weeks (301 days), mean (SD)
Severe ongoing	0.1% (1)	1% (2)	Severe ongoing: pp. ≈ 1	6.9 (.16)	8.1 (1.6)	250 (88)
Moderate ongoing	0.1% (1)	0	Moderate ongoing daily: pp. ≈ 1	7 (0)	4.2 (0)	301 (0)
Mild ongoing	0.1% (1)	0.4% (1)	Moderate ongoing not daily: pp. = .5 Moderate ongoing daily: pp. = .5	6.1 (.31)	3.0 (1.1)	200 (76)
Minor ongoing/recovered	16% (133)	8% (22)	Recovery: pp. = .97/Late recovery: pp. = .18	2.2 (2.1)	1.3 (.4)	2 (22)
Severe fluctuating	2% (15)	11% (28)	Severe ongoing: pp. = .77 Moderate ongoing not daily: pp. = .19	6.1 (1.3)	7.3 (.9)	239 (61)
Moderate fluctuating	6% (47)	15% (40)	Moderate ongoing not daily: pp. = .57 Moderate ongoing daily: pp. = .36	5.2 (1.5)	5.1 (.6)	206 (69)
Mild fluctuating	9% (75)	14% (38)	Moderate ongoing not daily: pp. = .62 Moderate ongoing daily: pp. = .24 Improvement w/ relapse: pp. = .13	4.0 (1.7)	3.2 (.7)	155 (81)
Minor fluctuating	2% (19)	1% (3)	Moderate ongoing not daily: pp. = .41 Improved w/ relapse: pp. = .31 Moderate ongoing daily: pp. = .23	3.9 (2.1)	1.8 (.5)	143 (90)
Severe episodic	24% (196)	28% (74)	Mild episodic: pp. = .31 Improvement w/ relapse: pp. = .25 Slow improvement: pp. = 0.23	3.3 (1.3)	4.2 (1.2)	51 (44)
Moderate episodic	16% (127)	14% (36)	Mild episodic: pp. = .42 Slow improvement: pp. = .19 Late recovery: pp. = .15 Improvement w/ relapse: pp. = .11	2.6 (1.2)	3.0 (.7)	29 (28)
Mild episodic	13% (104)	3% (7)	Mild episodic: pp. = .40 Late recovery: pp. = .26/Recovery: pp. = .14 Slow improvement: pp. = .12	1.9 (1.1)	1.9 (.5)	19 (26)
Minor episodic	0.6% (5)	1% (3)	Late recovery: pp. = .50/Recovery: pp. = .25 Improvement w/ relapse: pp. = .13Mild episodic: pp. = .12	1.2 (.4)	1 (0)	6 (7)
Severe single episode	1.8% (15)	1% (3)	Recovery: pp. = .87	4.3 (1.9)	6.4 (1.0)	6 (4)
Moderate single episode	2% (20)	1% (3)	Recovery: pp. = .83 /Late recovery: pp. = .13	2.4 (1.2)	4.4 (.6)	3 (2)
Mild single episode	6% (46)	1% (3)	Recovery: pp. = .55/Late recovery: pp. = .45	1.8 (1.0)	2.5 (.5)	2 (2)
Minor single episode	1% (9)	0	Recovery: pp. = .56/Late recovery: pp. = .44	1.1 (.2)	1 (0)	1 (1)

*LCA* Latent Class Analysis

^a^Mean probability of patients in the trajectory subgroup belonging to each of the previously identified LCA derived trajectory patterns. Patterns with posterior probability <0.1 were not listed

The ‘minor ongoing/recovered subgroup’, ‘minor episodic subgroup’ and four subgroups of minor to severe ‘single episodes’ were quite similar with very few LBP days and high probabilities of belonging to LCA patterns of recovery (Table 4). These could be reconceptualised as being one broad subgroup of minor LBP. Also, ‘mild fluctuating LBP’ and ‘moderate fluctuating LBP’ had quite similar profiles and distinguishing between these subgroups may be irrelevant for some purposes.

### Comparison between classification using the defined trajectory subgroups and using the LCA-derived trajectory patterns

There was a clear relationship between the defined trajectory subgroups and most of the LCA-derived trajectory patterns. This is reflected in the probabilities of people within each of the defined trajectory subgroups belonging to particular LCA-derived trajectory patterns, showing that in most subgroups, people were very likely to belong to one or two of the eight LCA patterns (Table 4). For example, people in the subgroups ‘minor ongoing’, ‘minor episodic’, ‘mild single episode’, and ‘minor single episode’ had high probabilities (ranging from 0.75 to 1.00) of belonging to the *recovered* trajectory patterns.

For the ‘minor fluctuating LBP’ subgroup and the subgroups with mild to severe ‘episodic LBP’, the relationship to the LCA-derived trajectory patterns was less clear. This appeared to be caused by the subgroup definitions not being designed to differentiate *improvement with relapse* (a pattern identified by LCA) from episodic or fluctuating pain, and also not differentiating between daily and non-daily pain. The apparent mismatch that people in the ‘severe episodic LBP subgroup’ were likely to be in a trajectory pattern of *mild episodic pain* was due to the subgroup definition being based on the severity within the worst week, whereas the trajectory pattern was labelled according to the average course being mild. The ‘severe ongoing LBP subgroup’ had a very low prevalence in these samples and people classified as having the *severe ongoing* LCA-derived trajectory pattern were classified into the ‘severe fluctuating LBP subgroup’. This was caused by the defined trajectory subgroups being named *fluctuating* if an individual’s pain intensity exceeded their mean pain +/− one point on a 0–10 scale, while the LCA-derived trajectory pattern was labelled *ongoing* despite some fluctuations in pain intensity.

### Characteristics of fluctuations and episodes

Fluctuations were present in 83% of the sample with a median of 6 (IQR 2–16) weeks containing deviations of one point or more from the mean intensity. In the weeks with fluctuations, the median deviation from the mean pain intensity was 2.3 (IQR 1.8–3.0) points (range 1.0 to 8.7). Among people in the ‘fluctuating LBP’ subgroups, 82% had at least one fluctuation that deviated two or more points from the mean, which illustrates that a change in criteria for fluctuations from +/−1 point to +/−2 points would not change the classification of most people in these subgroups.

Pain-free periods of any length were reported by 82% of people who were not completely pain-free, such periods occurred a median of 3 (IQR 1–5) times (range 0–15 times) during 43 weeks and lasted a median of 2 (IQR 1–6) weeks (range 1–42). Thirty-four percent of the pain-free periods lasted only 1 week and 61% lasted one to 3 weeks. In light of the recommended use of the threshold of four pain-free weeks as a marker of separation between episodes, it is noteworthy that the average pain intensity and number of days with LBP in the week after a pain-free period did not differ between pain-free periods that ranged from one week to >20 weeks (Table 5).

**Table 5:** LBP intensity and frequency in the first week following a pain-free period of from 1 to 20+ weeks

Duration of pain-free period, weeks	Mean (SD) pain intensity in the first week after a pain-free period, 0–10 NRS	Mean (SD) number of days with LBP in the first week after a pain-free period	n
1	3.35 (1.9)	2.37 (1.5)	948
2	3.32 (1.8)	2.26 (1.4)	479
3	3.43 (1.9)	2.31 (1.5)	305
4	3.30 (1.8)	2.05 (1.4)	192
5	3.33 (1.8)	2.22 (1.5)	153
6	3.49 (1.9)	2.34 (1.6)	132
7	3.35 (2.0)	2.11 (1.3)	104
8	3.36 (2.1)	2.27 (1.6)	67
9	3.48 (2.1)	2.28 (1.5)	69
10–15	3.32 (1.9)	2.48 (1.8)	243
15–20	3.71 (2.1)	2.45 (1.6)	100
>20	3.67 (2.0)	3.19 (2.1)	228

### Comparison between episodic and fluctuating LBP

**Fig. 4:**
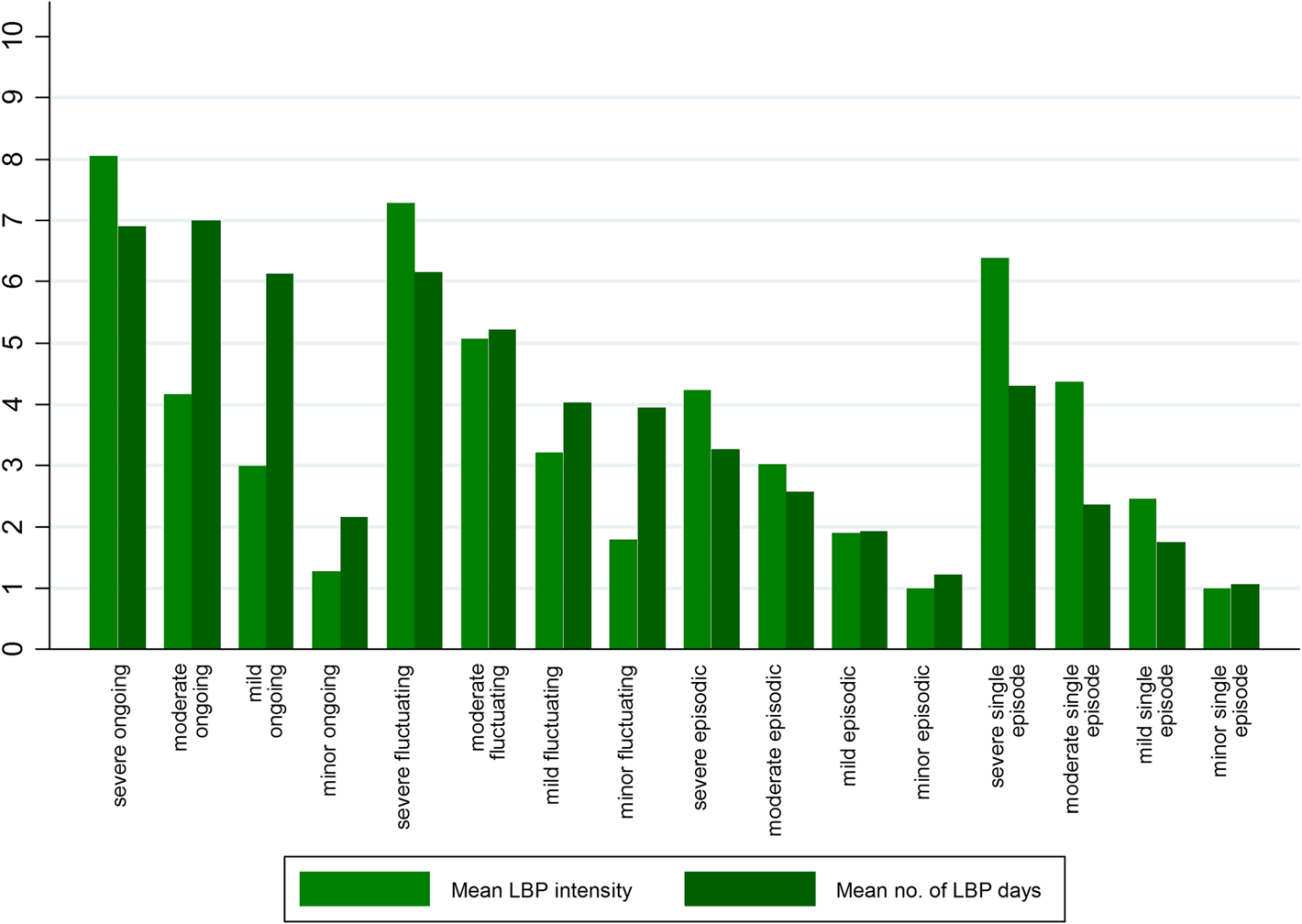
LBP intensity and frequency in the LBP trajectory subgroups in weeks when any LBP was reported

LBP occurred on more days during the week in weeks with any pain in the fluctuating pain subgroups than in the episodic subgroups, and pain was also more intense when present in fluctuating subgroups than in episodic subgroups (Table 4 and Fig. 4). When comparing the episodic and fluctuating defined trajectory subgroups that had the same levels of LBP intensity (severe, moderate, mild, minor), the fluctuating subgroups were significantly more distressed at baseline, had more intense leg pain at baseline, higher levels of activity limitation after 3 months, and more negative expectations about future LBP after 12 months (Table 6).

**Table 6:** Comparison of patients with fluctuating and episodic LBP trajectory patterns

	Fluctuating	Episodic
Mean distress at baseline (SD)		
Severe*	17.6 (8.8)	9.4 (7.8)
Moderate*	13.0 (9.3)	7.7 (6.9)
Mild*	8.8 (7.8)	6.1 (5.6)
Minor (NS)	8.6 (7.3)	7.8 (.8)
Mean leg pain intensity at baseline (SD)		
Severe*	5.9 (3.1)	2.7 (3.0)
Moderate*	3.5 (3.0)	2.5 (2.8)
Mild*	3.3 (3.0)	1.9 (2.4)
Minor (NS)	2.1 (2.3)	2.9 (3.2)
Mean activity limitation at 3-month follow-up (SD)		
Severe*	61.6 (19.3)	19.8 (21.8)
Moderate*	49.5 (24.1)	10.3 (13.8)
Mild*	29.6 (21.5)	6.1 (8.2)
Minor (NS)	19.9 (22.7)	7.6 (15.2)
Expectations of future LBP at 12-month follow-up (%)		
Severe*	*n* = 27	*n* = 235
No future LBP	0	7%
Episodic mild	0	53%
Ongoing mild	7%	9%
Episodic – varying intensity	11%	24%
Ongoing – varying intensity	37%	2%
Episodic severe	19%	5%
Ongoing severe	26%	0
Moderate*	*n* = 72	*n* = 146
No future LBP	1%	12%
Episodic mild	15%	67%
Ongoing mild	31%	5%
Episodic – varying intensity	22%	14%
Ongoing – varying intensity	22%	2%
Episodic severe	8%	0
Ongoing severe	0	0
Mild*	*n* = 80	*n* = 134
No future LBP	3%	18%
Episodic mild	25%	62%
Ongoing mild	29%	8%
Episodic – varying intensity	33%	11%
Ongoing – varying intensity	6%	0
Episodic severe	3%	1%
Ongoing severe	3%	0
Minor (NS)	*n* = 20	*n* = 13
No future LBP	5%	8%
Episodic mild	50%	85%
Ongoing mild	20%	0
Episodic – varying intensity	15%	8%
Ongoing – varying intensity	5%	0
Episodic severe	5%	0
Ongoing severe	0	0

^*^ *p < .01*

The sensitivity analysis revealed that these group differences were not altered substantially by considering LBP to be ‘episodic’ if a pain free period of 2 weeks or more was present instead of requiring a pain free period of at least 4 weeks. This change in definition mainly shifted people from the ‘mild fluctuating subgroup’ (*n* = 37/113) and ‘minor fluctuating subgroup’ (*n* = 11/22) LBP to ‘episodic’ subgroups.

## Discussion

In this study, we classified the LBP trajectories of samples of patients using previously recommended definitions for defined trajectory subgroups and found that data from weekly measures of LBP did allow us to categorise every participant into these subgroup definitions, which demonstrated that the definitions were readily applicable to these data. Furthermore, we explored how individuals’ classification into defined trajectory subgroups matched with their membership of previously identified LCA-derived trajectory patterns, and the results showed a strong match for most subgroups.

On the basis of our results, we suggest that the applied trajectory subgroup definitions might benefit from further refinement as the trajectories of this sample seemed to be adequately described by fewer subgroups than the 16 previously defined. Firstly, because almost nobody fulfilled the criteria for the ‘ongoing LBP subgroups’, at least in this cohort, and secondly, because very consistent pain intensities may not be substantially different from pain intensities that fluctuate at the level of +/− one point on an 11-point scale. Almost all people in this cohort had these small fluctuations and it is possible that this just reflects measurement error [21]. The question of what magnitude of fluctuations is a useful differentiator from those people experiencing constant levels of pain remains to be investigated, but ‘ongoing’ and ‘fluctuating’ subgroups might be collectively considered to be ‘ongoing LBP with or without fluctuations’. Also, we observed that subgroups with minor LBP and subgroups with single episodes should probably be considered one subgroup. In contrast, the value of distinguishing between fluctuating and episodic LBP was clearly supported by the observation that episodic LBP was more benign (less intense and fewer days of LBP per week, less distress, and less activity limitation) than fluctuating LBP, and people with episodic LBP had more positive expectations about future LBP than people with fluctuating LBP. However, the large subgroup of ‘severe episodic’ pain was somewhat heterogeneous and people in that group who reported very few days with LBP may fit better with the ‘single episodes’ subgroups.

We defined episodic pain as LBP recurring after a period of at least four pain-free weeks, which was based on previous recommendations [19, 20]. We found that LBP reoccurring after pain-free periods of four or more weeks was no different from LBP reoccurring after one to 3 weeks and in that sense, our data do not provide support for four pain-free weeks indicating an empirically distinct threshold. However, it should also be noted that our data also did not suggest that shorter or longer pain-free periods would provide a better definition of ‘episodic’, and it is noteworthy that when using the recommended definition of four or more weeks, the ‘episodic subgroups’ were distinctly different on other pain characteristics from non-episodic LBP subgroups, which adds support to their construct validity. Across subgroups, most people had periods without pain and therefore short-lasting reports of being pain-free do not appear to be a suitable basis for defining recovery from LBP.

Demonstrating that the previously suggested defined trajectory subgroups can be applied to data and that all trajectories in this cohort could be unambiguously classified suggests that it is practical to use these definitions to identify LBP trajectory subgroups in new cohorts. The availability of operationally defined subgroups that allow the classification of trajectories of individuals, establishes a potential method for directly comparing the trajectory subgroups across cohorts without the need for the statistical identification of latent classes in each cohort. Furthermore, the development of exact criteria for defined trajectory subgroups may allow identification of homogenous LBP subgroups, for example, as an inclusion criterion for intervention studies. However, this would require that the LBP trajectory of individuals could be established prior to inclusion or that people are able to identify what trajectory subgroup they belong to based on recall [22].

For further refinement of the criteria for defined trajectory subgroups, it would be useful to explore if ongoing and fluctuating LBP are substantially different or could be considered one type of pain variation. This would require cohorts with a higher prevalence of ongoing LBP. One aspect of this is that we need better insight into what magnitude of pain fluctuations may be of importance. We operationalised the definition of fluctuations as a deviation of +/−1 point from the mean. This small variation may actually simply be ‘noise’ or measurement error. However, eight in ten people with these small fluctuations also had larger variations in pain intensity (at least +/−2 points from the mean). Also, additional studies should investigate if the subgroups of ‘minor LBP’ and ‘single episode LBP’ are generally experienced differently by people or could also be considered one phenotype. This may include looking at single episodes of LBP lasting more than 2 weeks, as these were grouped as ‘episodic LBP’ using the current definitions.

While there is appeal in operationalising one fixed approach to trajectory subgrouping, the level of detail needed to differentiate between subgroups may not be the same for all purposes. For example, there may be different requirements if studying trajectory subgroups as potential treatment effect modifiers than if using the subgroups for patient education [8]. Furthermore, there is a need for determining if there is a useful way to apply modified subgroup definitions if data is collected less frequently than weekly.

The strengths of this study were the availability of weekly collected data about both LBP intensity and frequency in two different patient samples [13]. Also, it was advantageous that the LCA-derived trajectory patterns had been identified in the samples before the subgroup definitions were made, as the choice of a LCA model involves some subjective decisions. In contrast, it may be a weakness that the LCA patterns identified from these samples were included in one of the nine studies that informed the development of the subgroups definitions, but due to the number of studies, this is likely to have not been a large influence [8]. One limitation of this study was that only people with relatively complete data were classified, as we excluded the 13% of the cohort who had many missing values. We did so because these could not be unambiguously subgrouped without imputing data and, in this initial study, we opted to use only observed data. Depending on the purpose of subgrouping, imputation may be appropriate. We chose to exclude data from the first 9 weeks after care- seeking to make the results more widely relevant and to reduce the number of defined trajectory subgroups that were explored. However, for some purposes, the inclusion of early change patterns could be relevant and could be applied by using similar principles to those presented here.

## Conclusion

This study was the first to demonstrate that suggested definitions of LBP trajectory subgroups can be readily applied to individuals’ observed data resulting in subgroups that match well with LCA-derived trajectory patterns. We suggest that the number of trajectory subgroups can be reduced by merging some subgroups with infrequent and mild LBP. Further, we suggest that minor fluctuations in pain intensity might be conceptualised as ‘ongoing LBP’. Lastly, we found clear support for distinguishing between fluctuating and episodic LBP.

## Abbreviations

IQR: Inter Quartile Range
LBP: Low Back Pain
LCA: Latent Class Analysis
PP: Posterior Probability
SD: Standard Deviation

## References

[1] Axen I, Leboeuf-Yde C (2013). Trajectories of low back pain. Best Pract Res Clin Rheumatol.

[2] Downie AS, Hancock MJ, Rzewuska M, Williams CM, Lin CC, Maher CG. Trajectories of acute low back pain: a latent class growth analysis. PAIN. 2016;157(1):225–34. 10.1097/j.pain.0000000000000351 26397929

[3] Dunn KM, Campbell P, Jordan KP (2013). Long-term trajectories of back pain: cohort study with 7-year follow-up. BMJ Open.

[4] Kongsted A, Kent P, Hestbaek L, Vach W. Patients with low back pain had distinct clinical course patterns that were typically neither complete recovery nor constant pain. A Latent Class Analysis of longitudinal data. Spine J. 2015;15(5):885-94. 10.1016/j.spinee.2015.02.012 25681230

[5] Macedo LG, Maher CG, Latimer J, McAuley JH, Hodges PW, Rogers WT (2014). Nature and determinants of the course of chronic low back pain over a 12-month period: a cluster analysis. Phys Ther.

[6] Deyo RA, Bryan M, Comstock BA, Turner JA, Heagerty P, Friedly J, Avins AL, Nedeljkovic SS, Nerenz DR, Jarvik JG. Trajectories of Symptoms and Function in Older Adults with Low Back Disorders. Spine (Phila Pa 1976). 2015;40(17):1352-362. 10.1097/BRS.0000000000000975 25996537

[7] Chen C, Hogg-Johnson S, Smith P (2007). The recovery patterns of back pain among workers with compensated occupational back injuries. Occup Environ Med.

[8] Kongsted A, Kent P, Axen I, Downie AS, Dunn KM (2016). What have we learned from ten years of trajectory research in low back pain?. BMC Musculoskelet Disord.

[9] Kent P, Keating JL, Leboeuf-Yde C (2010). Research methods for subgrouping low back pain. BMC Med Res Methodol.

[10] Foster NE, Hill JC, Hay EM (2011). Subgrouping patients with low back pain in primary care: are we getting any better at it?. ManTher.

[11] Hagenaars JA, AL MC (2002). Applied latent class analysis.

[12] Kongsted A, Nielsen AM (2017). Latent class analysis in health research. J Phys.

[13] Hestbaek L, Munck A, Hartvigsen L, Jarbol DE, Sondergaard J, Kongsted A (2014). Low back pain in primary care: a description of 1250 patients with low back pain in danish general and chiropractic practice. Int J Family Med.

[14] Kongsted A, Vach W, Axo M, Bech RN, Hestbaek L (2014). Expectation of recovery from low back pain: a longitudinal cohort study investigating patient characteristics related to expectations and the association between expectations and 3-month outcome. Spine (Phila Pa 1976).

[15] Kongsted A, Andersen CH, Hansen MM, Hestbaek L (2016). Prediction of outcome in patients with low back pain - a prospective cohort study comparing clinicians' predictions with those of the start back tool. Man Ther.

[16] Bech P, Rasmussen NA, Olsen LR, Noerholm V, Abildgaard W (2001). The sensitivity and specificity of the major depression inventory, using the present state examination as the index of diagnostic validity. J Affect Disord.

[17] Kent P, Lauridsen HH (2011). Managing missing scores on the Roland Morris disability questionnaire. Spine (Phila Pa 1976).

[18] Albert HB, Jensen AM, Dahl D, Rasmussen MN (2003). Criteria validation of the Roland Morris questionnaire. A Danish translation of the international scale for the assessment of functional level in patients with low back pain and sciatica. Ugeskr Laeger.

[19] Stanton TR, Latimer J, Maher CG, Hancock MJ (2011). A modified Delphi approach to standardize low back pain recurrence terminology. Eur Spine J.

[20] de Vet HC, Heymans MW, Dunn KM, Pope DP, van der Beek AJ, Macfarlane GJ, Bouter LM, Croft PR (2002). Episodes of low back pain: a proposal for uniform definitions to be used in research. Spine (Phila Pa 1976).

[21] Childs JD, Piva SR, Fritz JM (2005). Responsiveness of the numeric pain rating scale in patients with low back pain. Spine.

[22] Dunn KM, Campbell P, Jordan KP: Visual trajectories: can people tell you their back pain trajectory? In: XIII International Back Pain Forum. 2014; Campos do Jordao, Brazil; 2014.

[23] Act on Research Ethics Review of Health Research Projects [http://www.nvk.dk/english/act-on-research]. Accessed 29 June 2017.

